# Sulfoxide‐Containing Polymer‐Coated Nanoparticles Demonstrate Minimal Protein Fouling and Improved Blood Circulation

**DOI:** 10.1002/advs.202000406

**Published:** 2020-05-17

**Authors:** Ruirui Qiao, Changkui Fu, Yuhuan Li, Xiaole Qi, Dalong Ni, Aparna Nandakumar, Ghizal Siddiqui, Haiyan Wang, Zheng Zhang, Tingting Wu, Jian Zhong, Shi‐Yang Tang, Shuaijun Pan, Cheng Zhang, Michael R. Whittaker, Jonathan W. Engle, Darren J. Creek, Frank Caruso, Pu Chun Ke, Weibo Cai, Andrew K. Whittaker, Thomas P. Davis

**Affiliations:** ^1^ ARC Centre of Excellence in Convergent Bio‐Nano Science and Technology and Australian Institute for Bioengineering and Nanotechnology The University of Queensland Brisbane QLD 4072 Australia; ^2^ ARC Centre of Excellence in Convergent Bio‐Nano Science and Technology Monash Institute of Pharmaceutical Sciences Monash University 381 Royal Parade Parkville VIC 3052 Australia; ^3^ Key Laboratory of Modern Chinese Medicines China Pharmaceutical University Nanjing 210009 China; ^4^ Departments of Radiology and Medical Physics University of Wisconsin – Madison Madison WI 53705 USA; ^5^ Monash Institute of Pharmaceutical Sciences Monash University 381 Royal Parade Parkville VIC 3052 Australia; ^6^ Institute for Hepatology National Clinical Research Center for Infectious Disease Shenzhen Third People's Hospital Guangdong Province Shenzhen 518112 China; ^7^ College of Food Science & Technology Shanghai Ocean University Shanghai 201306 China; ^8^ Department of Electronic Electrical and Systems Engineering School of Engineering University of Birmingham Edgbaston Birmingham B15 2TT UK; ^9^ ARC Centre of Excellence in Convergent Bio‐Nano Science and Technology and the Department of Chemical Engineering The University of Melbourne Parkville Victoria 3010 Australia

**Keywords:** long circulation, low‐fouling, nanoparticles, sulfoxide‐containing polymers

## Abstract

Minimizing the interaction of nanomedicines with the mononuclear phagocytic system (MPS) is a critical challenge for their clinical translation. Conjugating polyethylene glycol (PEG) to nanomedicines is regarded as an effective approach to reducing the sequestration of nanomedicines by the MPS. However, recent concerns about the immunogenicity of PEG highlight the demand of alternative low‐fouling polymers as innovative coating materials for nanoparticles. Herein, a highly hydrophilic sulfoxide‐containing polymer—poly(2‐(methylsulfinyl)ethyl acrylate) (PMSEA)—is used for the surface coating of iron oxide nanoparticles (IONPs). It is found that the PMSEA polymer coated IONPs have a more hydrophilic surface than their PEGylated counterparts, and demonstrate remarkably reduced macrophage cellular uptake and much less association with human plasma proteins. In vivo study of biodistribution and pharmacokinetics further reveals a much‐extended blood circulation (≈2.5 times longer in terms of elimination half‐life *t*_1/2_) and reduced accumulation (approximately two times less) in the organs such as the liver and spleen for IONPs coated by PMSEA than those by PEG. It is envisaged that the highly hydrophilic sulfoxide‐containing polymers have huge potential to be employed as an advantageous alternative to PEG for the surface functionalization of a variety of nanoparticles for long circulation and improved delivery.

## Graphical abstract

An innovative class of sulfoxide‐containing polymer is used as an exceptional surface functionalization material for nanoparticles. The highly hydrophilic nature of the polymer endows nanoparticles with remarkable low‐fouling property as demonstrated by weak interaction with plasma proteins, prolonged blood circulation, and improved biodistribution profile of conjugated nanoparticles.

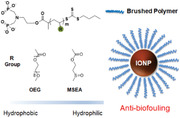

## 1 Introduction

Nanomedicines offer unique features for the treatment of human diseases such as cancer.^[^ ^1^ ^]^ With the possibility of engineering nanoparticles with tailored physicochemical properties and biological/medical functions, nanomedicines are regarded as a new paradigm to overcome intrinsic limitations of conventional therapies, leading to more effective and safer disease treatment.^[^ ^2^ ^]^ However, despite much progress in fundamental research, clinical translation of nanomedicine faces significant challenges.^[^ ^3^ ^]^ Specifically, most nanomedicines have not progressed past Phase II clinical trials due to failure to achieve anticipated therapeutic effects.^[^ ^4^ ^]^ This is largely due to a low delivery efficiency of nanomedicine to diseased tissue caused by biological barriers, including the mononuclear phagocytic system (MPS) in particular.^[^ ^5^ ^]^ Only a small proportion of dosed nanomedicines are able to reach the target site, while the majority of nanomedicines (>95%) are found to accumulate mainly within the liver and spleen, organs contributing largely to the MPS.^[^ ^5^, ^6^ ^]^ Thus, minimizing the interaction of nanomedicines with the MPS to increase their blood circulation time represents an effective approach to improving the delivery efficiency of nanomedicines, a vital element toward their clinical translation.

Conjugation of polyethylene glycol (PEG) on the surface of nanomedicines has often been used to impart so‐called “stealth” properties.^[^ ^7^ ^]^ The hydrophilicity and large conformational freedom of the PEG chain reduces the interaction of nanomedicines with biomolecules that possibly facilitate recognition by the immune system, and hence minimize their uptake by the MPS, leading to extended circulation in the bloodstream. However, recent studies have revealed that the intrinsic amphiphilic nature of PEG can also facilitate nonspecific interaction of PEGylated entities with proteins, contributing to unwanted recognition and further sequestration by the immune system.^[^ ^8^ ^]^ As a result, it could significantly shorten the blood circulation time of nanoparticles and compromise the function of PEGylation. Consequently, this has driven the development of alternative hydrophilic polymers as new low‐fouling materials for surface coating of nanoparticles.^[^ ^9^ ^]^ To date, a variety of hydrophilic polymers including but not limited to poly(amino acid)s, poly(vinyl pyrrolidone), poly(glycerol), polybetaines (zwitterionic polymers), poly(2‐oxazoline)s, poly(acrylamide), poly(*N*‐(2‐hydroxypropyl)methacrylamide), and glycopolymers have been investigated as possible alternatives to PEG for different biomedical uses.^[^ ^9c^ ^]^ In particular, the polybetaine‐based zwitterionic polymers demonstrate appealing low‐fouling property and have been extensively used for diverse biological applications.^[^ ^10^ ^]^ Despite this, many of these polymers typically show higher levels of bio‐fouling in comparison to PEG. On the other hand, the chemical nature of polymers significantly influences their interaction with biological system.^[^ ^11^ ^]^ Polymers that are more hydrophilic and resistant to protein binding/association are expected to circulate longer in the bloodstream and accumulate less in the MPS organs.^[^ ^12^ ^]^ Such polymers are in urgent demand as promising alternatives to PEG for nanoparticles coating to achieve enhanced pharmacokinetics and therapeutic outcomes.

The polar sulfoxide group has been increasingly used as a useful structural element to promote hydrophilicity of various functional polymers. These sulfoxide‐containing polymers have demonstrated the potential to enable broad applications such as molecular imaging and drug delivery.^[^ ^13^ ^]^ In particular, a sulfoxide‐containing polymer, poly(2‐(methylsulfinyl)ethyl acrylate) (PMSEA), has been recently examined, displaying high hydrophilicity and outstanding water solubility.^[^ ^14^ ^]^ Accordingly, PMSEA has been used to alter the relaxation properties and improve greatly the imaging sensitivity of magnetic resonance imaging (MRI) contrast agents.^[^ ^15^ ^]^ We hypothesized that this innovative PMSEA polymer with a highly hydrophilic nature may act as an exceptional low‐fouling coating material for nanoparticles to confer a superior stealth property. In this work, we aimed to investigate the effect of grafting PMSEA to the surface of iron oxide nanoparticles (IONPs) on the cellular uptake and in vivo behavior of the nanoparticles. Moreover, we conducted point‐to‐point comparisons between PMSEA‐coated and PEGylated nanoparticles on their physical and biological properties. Our results highlight that the utilization of PMSEA can impart superior low‐fouling property to nanoparticles in comparison to PEG counterpart. PMSEA‐coated nanoparticles demonstrated much reduced interaction with protein molecules, and exhibited longer blood circulation time and reduced MPS accumulation compared with PEGylated nanoparticles, which would contribute significantly to improving the efficiency of nanoparticles in systemic delivery to the region of interest.

## 2 Results and Discussion

### 2.1 Iron Oxide Nanoparticles as Model Nanoparticles

Iron Oxide Nanoparticles (IONPs) have been frequently investigated as MRI contrast agents due to their unique physical and chemical properties, for example, superparamagnetic or paramagnetic properties. In the past two decades, tremendous efforts have been made to improve the synthesis of IONPs and now particles with high crystallinity, controlled size, and size distribution can be obtained through the thermal decomposition method.^[^ ^16^ ^]^ Herein, we used oleic acid (OA)‐coated Fe_3_O_4_ nanoparticles to investigate the effects of different polymer coatings on their‐induced biological responses.

### 2.2 Chemical Design and Synthesis of Macromolecular Surface Ligands for IONPs

**Figure 1:**
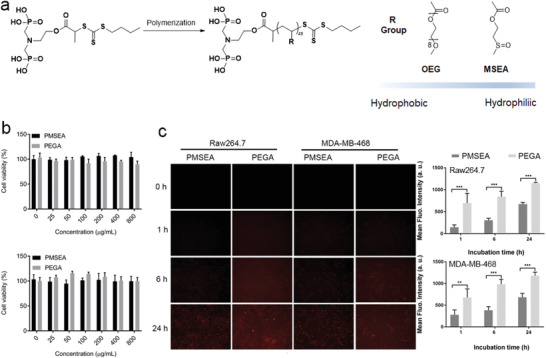
a) Synthesis of brushed polymer through RAFT polymerization; b) Cytotoxicity of PMSEA and PEGA polymers on Raw 264.7 and MDA‐MB‐468 cell lines; c) Cellular uptake of PMSEA and PEGA polymers on Raw 264.7 and MDA‐MB‐468 cell lines.

The chemical design of the IONP macromolecular ligand was based on a brush polymeric structure which combined a terminal diphosphate group for anchoring to Fe atom at the surface of the IONPs, and a biocompatible brush polymer composed of repeating units of either 2‐(methylsulfinyl)ethyl acrylate (MSEA) or oligo(ethylene glycol) methyl monoether acrylate (OEGA). As shown in **Figure** **1**, the polymers were synthesized through reversible addition−fragmentation chain‐transfer (RAFT) polymerization using a diphosphonate‐terminated chain transfer agent (CTA). The detailed synthesis and characterization of the CTA is provided in Figure S1, Supporting Information. Comparing to the linear‐structured polymers, the side‐chain polymers prepared through RAFT polymerization offered a more feasible approach for the synthesis of multi‐functional polymers through easy incorporation of groups, which endows surface engineering of nanoparticles with robust metal coordination ability, biocompatibility as well as functionalization ability.^[^ ^9a^ ^]^

As shown in Figures S2 and S3, Supporting Information, PMSEA and Poly oligo(ethylene glycol) methyl monoether acrylate (PEGA) polymers with a similar degree of polymerization (DP) were thoroughly characterized by ^1^H NMR and size exclusion chromatography (SEC) (Figure S4, Supporting Information). The molecular information of the polymers is summarized in Table S1, Supporting Information. The as‐synthesized brush polymers are demonstrated with a similar DP and a narrow molecular weight distribution (PDI = 1.29), indicative of well‐controlled polymerizations by RAFT.

We first studied the cytotoxicity of PMSEA and PEGA polymers using two different cell lines—macrophage cell Raw 264.7 and breast cancer cell MDA‐MB‐468. As shown in Figure 1b, both polymers demonstrated no significant toxicity to the Raw 264.7 and MDA‐MB‐468 cells in a concentration range of 0–800 µg mL^−1^.

We next investigated the cellular uptake of PMSEA and PEGA polymers on same cell lines. To facilitate such study, the RAFT‐based polymers were labeled with a fluorescent dye Cy5 through a “click” reaction between the –SH group of aminolyzed polymers and maleimide‐terminated Cy5 (Figure S5, Supporting Information). Time‐dependent analyses of cellular uptake of polymer‐Cy5 were conducted. As shown in Figure 1c, both PMSEA and PEG polymers could be internalized by Raw 264.7 and MDA‐MB‐468 cells through endocytosis, including both pinocytosis and phagocytosis pathways.^[^ ^17^ ^]^ The PMSEA polymer demonstrated a much lower cell uptake by both Raw 264.7 and MDA‐MB‐468 cells in comparison to PEGA polymer, indicating the superior “stealth” behavior of PMSEA polymers.

### 2.3 Immunogenicity of PMSEA and PEGA Polymers

**Figure 2:**
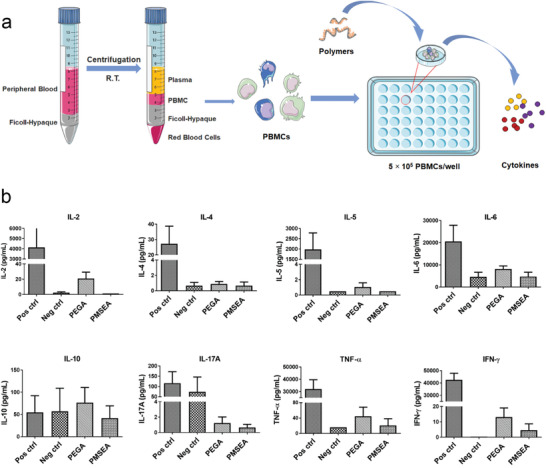
a) Illustration of the cytokine release experiment; b) Cytokines release from PBMC after incubating with PMSEA and PEGA polymers.

Before further grafting polymers to IONPs, the immunogenicity of the synthesized comb‐like PMSEA and PEGA polymers were evaluated using an in vitro model by incubating human peripheral blood mononuclear cells (PBMCs) in the presence of polymers at a concentration of 2 mg mL^−1^ (**Figure** **2**a). Phorbol myristate acetate (PMA, 50 ng mL^−1^)/ionomycin (250 ng mL^−1^) and cell culture medium were used as the positive and negative control respectively. A panel of cytokines released by the cells after incubation for 20 h were measured, including pro‐inflammatory cytokines (IL‐6, IL‐17A, and TNF‐*α*), TH1 and TH2‐type cytokines (IFN‐*γ*, IL‐2 and IL‐4, IL‐5, IL‐10). In general, both polymers induced insignificant cytokine release as shown in Figure 2b, indicating they elicited very minimal immune responses. However, the cells exposed to PMSEA polymers released relatively less amounts of all the investigated cytokines than the commonly used comb‐like PEGA, suggesting a lower immunogenic risk of the PMSEA polymer and its potential as superior low‐fouling coating material for nanoparticles.

### 2.4 Characterization of Polymer‐Coated IONPs

**Figure 3:**
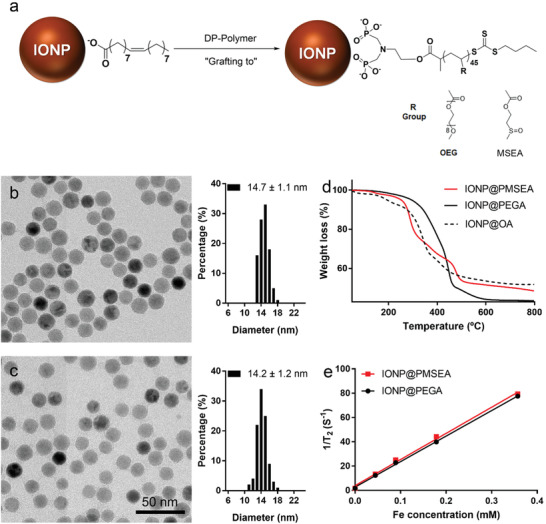
a) Surface grafting of IONP by diphosphonate‐terminated PMSEA and PEGA polymers, respectively; TEM and histogram of b) IONP@PMSEA; and c) IONP@PEGA; d) TGA analysis of IONP@PMSEA, IONP@PEGA and IONP@OA; e) *T*_2_ relaxation rate (*R*_2_) of IONP@PMSEA and IONP@PEGA in water against the concentration of Fe ion determined by 9.4 T MRI.

Diphosphonate‐terminated PMSEA and PEGA polymers‐coated IONPs were prepared using a “grafting to” approach featured by ligand exchange of the original hydrophilic surface coating of the IONP@OA to the biocompatible polymers (**Figure** **3**a). As displayed in Figure 3b,c, the particles (IONP@PMSEA and IONP@PEGA) were characterized by a monodisperse size of ≈14 nm and the particles did not display a size or shape change compared to their mother IONP@OA particles (Figure S6, Supporting Information).

We further obtained energy‐dispersive X‐ray spectroscopy (EDS) mapping to examine the distribution of iron and oxygen within the IONPs, as shown in Figure S7, Supporting Information. The EDS mapping indicates the presence of sulfur (S = O) attributed to the diphosphonate‐terminated PMSEA coating, indicating the successful grafting of PMSEA to IONPs (Figure S7a, Supporting Information). The amount of the polymer grafted on the nanoparticle surface was investigated by thermogravimetric analysis (TGA) and the weight loss was determined to be 51.5%, 56.5%, and 48.3% for IONP@PMSEA, IONP@PEGA and IONP@OA, respectively (Figure 3d). The TGA results revealed a similar surface coverage of IONPs by weight of the PMSEA and PEGA polymers. The superparamagnetic properties of both IONP@PMSEA and IONP@PEGA particles were investigated using a 9.4 T MRI. The transverse relaxivities *r*_2_ (efficiency to increase MRI contrast) were extracted from the linear regression fits of the experimental data, as shown in Figure 3e, and were determined to be 209.9 and 215.1 mm ^−1^s^−1^ for the PMSEA and PEG‐functionalized IONPs, respectively.

**Figure 4:**
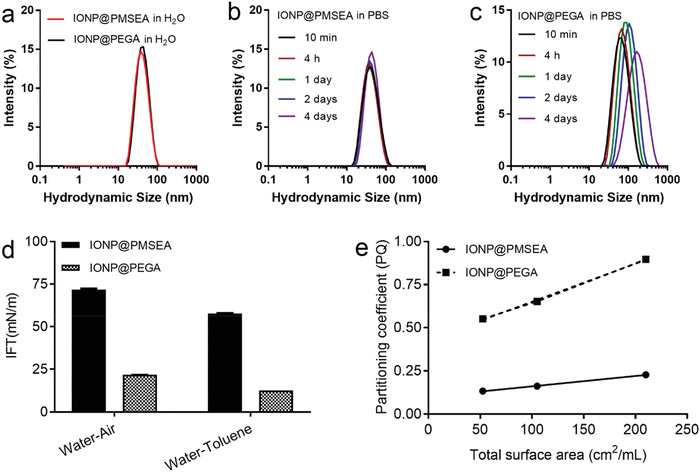
a) Hydrodynamic size distribution of IONP@PMSEA and IONP@PEGA in H_2_O, b) IONP@PMSEA, and c) IONP@PEGA in PBS buffer; d) Interfacial tension of IONP@PMSEA and IONP@PEGA nanoparticles, and e) partitioning quotient (PQ) of Rose Bengal.

In our previous work, we demonstrated the use of robust anchoring of the di/multi‐phosphonate‐ terminated PEGA polymers for the stabilization of IONPs in water and physiological buffers.^[^ ^9^, ^18^ ^]^ In the current work, we further evaluated the colloidal stability of the brush polymer grafted nanoparticles using dynamic light scattering (DLS). As shown in **Figure** **4**a, both IONP@ PMSEA and IONP@PEGA particles show comparable size (≈40 nm) with a narrow size distribution (PDI < 0.2). Additionally, these particles showed a long‐term stability in water following a more than one‐year storage at 4 °C. Of relevance to biomedical applications, the IONP@PMSEA nanoparticles were found to be more stable in phosphate buffered saline (PBS) compared with the IONP@PEGA particles, as indicated by the constant particle size and distribution over four‐day monitoring (Figure 4b,c).

Surface hydrophobicity of nanoparticles is regarded as a key factor for their interaction with plasma proteins and therefore has a strong influence on their in vivo fate.^[^ ^19^ ^]^ Nanoparticles with a more hydrophilic surface in the absence of nucleophilic or electrophilic groups can largely reduce the adsorption of the protein and therefore inhibit cellular uptake by the MPS.^[^ ^12a^ ^]^ The degree of nanoparticle hydrophobicity has also been reported to dedicate immune response both in vitro and in vivo.^[^ ^20^ ^]^ In the current study, we compared the hydrophobicity of the PMSEA‐ and PEGA‐grafted IONPs through time‐dependent pendant drop tensiometer measurements at the air–water and water–toluene interface. The equilibrium interfacial tension (IFT) was recorded through the time‐dependent dynamic surface tension plots, where the IFT approached an equilibrium value after a certain time (Figure S8, Supporting Information). As shown in Figure 4d, the IFT of the solution of IONP@PMSEA was significantly increased over that of the solution of IONP@PEGA particles, indicating a decreased hydrophobicity at the particle surface.^[^ ^12a^ ^]^ We further analyzed the surface hydrophobicity of IONPs by measuring the adsorption of a hydrophobic dye Rose Bengal to particles at increasing surface area.^[^ ^21^ ^]^ Rose Bengal undergoes partitioning between the particle surface and the dispersion medium, for example, water. For each particle concentration, the partitioning quotient (PQ) was calculated as

(1) $$\mathrm{PQ}\;=\frac{\mathrm{amount}\;\mathrm{of}\;\mathrm{Rose}\;\mathrm{Bengal}\;\mathrm{bound}\;\mathrm{on}\;\mathrm{surface}}{\mathrm{amount}\;\mathrm{of}\;\mathrm{Rose}\;\mathrm{Bengal}\;\mathrm{in}\;\mathrm{dispersion}\;\mathrm{medium}}$$

The PQ was plotted against the total surface area (TSA) of the nanoparticles, and the slope increased with increased surface hydrophobicity. The detailed calculation of TSA has been provided in Table S3, Supporting Information. As shown in Figure 4e, PMSEA‐grafted IONPs showed a much smaller slope than PEGA‐grafted IONPs. This indicates that the surface of IONP@PMSEA is more hydrophilic, resulting in a weaker adsorption of the Rose Bengal dye.

### 2.5 Protein Corona Analysis

**Figure 5:**
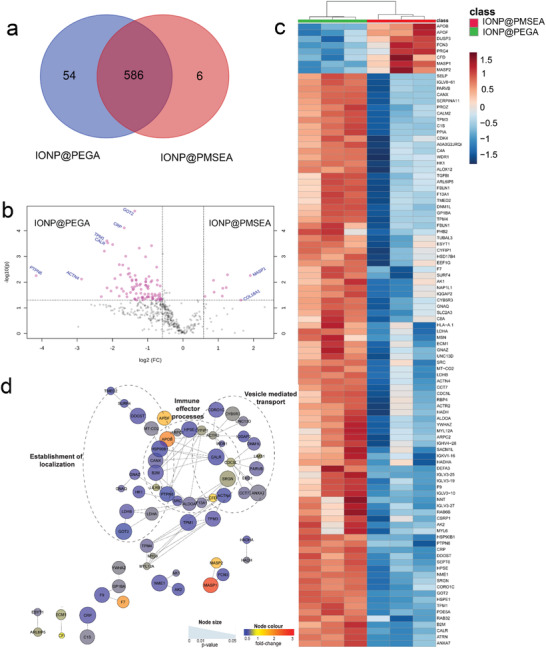
a) Venn diagram for the distribution of corona proteins associated with different IONPs after incubating in human plasma for 24 h; b) Volcano plot of differential protein abundance in IONP@PMSEA versus IONP@PEGA; c) Hierarchical clustering of IONP@PMSEA (red) and IONP@PEGA (green). Vertical clustering displays similarities between sample groups, while horizontal clusters reveal the relative abundances of the 101 most significantly different proteins. Proteins identified in at least two of three independent experiments, proteins above the significance threshold (*p*‐value *p*‐value < 0.05) and fold change ≥ 1.5 were considered significant. d) Network analysis of differentially regulated proteins in IONP@PMSEA versus IONP@PEGA. The network analysis was built using the STRINGdb interaction network analysis output (connectivity was based on experimental, database, and co‐expression evidence with a minimum interaction score of 0.7) in Cytoscape 3.6 with the ClusterONE algorithm. Node size represents *p*‐value and node color represents fold‐change from at least two of three independent replicates.

To determine the antifouling capacities of PEGA‐ and PMSEA‐grafted IONP, a proteomic study to identify the corona proteins of the nanoparticles was undertaken using label‐free liquid chromatography–mass spectrometry (LC‐MS)/MS analysis.^[^ ^22^ ^]^ A total of 646 proteins were detected in at least two out of the three replicates for the IONPs, of which 586 proteins were commonly seen in both samples (**Figure** **5**a). IONP@PEGA had the largest number of unique proteins enriched (54 proteins), whereas only six proteins were unique to IONP@PMSEA.

To identify the differences in corona protein abundance between IONP@PEGA and IONP@PMSEA, a differential analysis was conducted based on label free quantification of the proteins identified in both coronas. The vast majority of proteins appeared to be more abundant in the IONP@PEGA compared to IONP@PMSEA, seen as a negative log_2_ fold‐change (IONP@PMSEA/IONP@PEGA) in the volcano plot (Figure 5c). Statistical analysis revealed a total of 92 proteins were significantly more abundant in IONP@PEGA corona than in IONP@PMSEA, while only nine proteins were significantly more abundant in IONP@PMSEA (1.5‐fold difference, *p* ≤ 0.05) (Supplementary Excel sheet). Hierarchical clustering analysis and heatmap visualization of the relative abundance of the 101 significant proteins demonstrates the extensive and reproducible enrichment of proteins in the IONP@PEGA corona compared to IONP@PMSEA (Figure 5b). Network analysis of proteins that were significantly enriched in IONP@PEGA vs. IONP@PMSEA revealed that the IONP@PEGA corona has a unique proteomics signature characterized by significant enrichment of proteins involved in vesicle mediated transport (36 proteins), establishment of localization (48 proteins), and immune effector process (24 proteins) (Figure 5d). This comparative functional enrichment analysis of the corona proteins demonstrated that the proteins specifically associated with IONP@PEGA may be involved in nanoparticle transport, localization, and immune responses, whereas the proteins specifically associated with IONP@PMSEA had no significant enrichment of major cellular pathways.

### 2.6 In Vitro Cell Association Behavior of PMSEA‐Grafted IONPs

**Figure 6:**
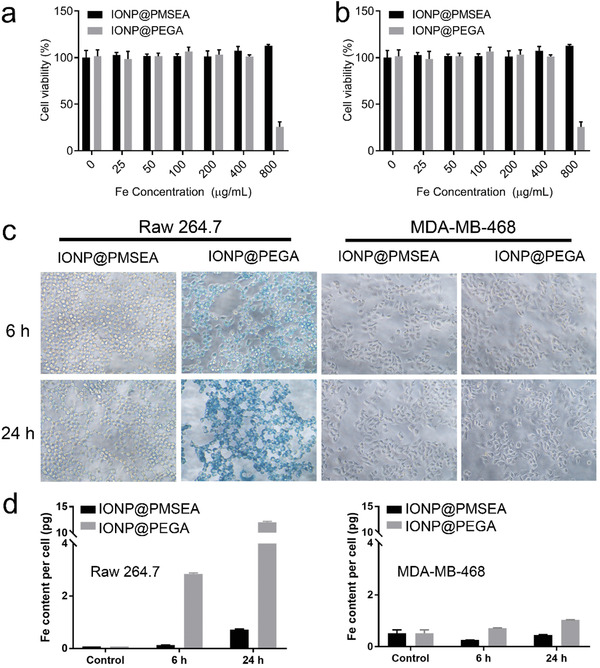
Cytotoxicity of IONP@PMSEA, IONP@PEGA on a) Raw264.7 and b) MDA‐MB‐468 cell lines after 24 h of incubation. c) Prussian Blue Staining of Raw 264.7 and MDA‐MB‐468 incubated with IONP@PMSEA and IONP@PEGA in serum‐containing cell culture medium; d) Quantitative analysis of IONP uptake on Raw 264.7 (left) and MDA‐MB‐468 (right) cell lines using ICP‐OES.

We first assessed the biosafety of the PMSEA‐grafted IONPs through the Alarma Blue assay using Raw 264.7 and MDA‐MB‐468 cell line (**Figure** **6**a,b). The two types of IONPs had a similar level of surface coating (≈50% surface coating from TGA results), and showed a negligible effect on the viability of cells up to an iron concentration of 200 µg mL^−1^. However, the PEGA‐coated IONPs showed an increased cytotoxicity to macrophages at the concentration of 800 µg mL^−1^, whereas the PMSEA‐coated IONPs were safe in all the cell lines used at this concentration. The observation of the toxicity of PEGylated‐IONPs at high concentrations can be attributed to the higher uptake (shows below) of Fe compared with the PMSEA‐coated counterparts, leading to an enhanced generation of reactive oxygen species (ROS) and ferroptosis. A similar effect was observed on the tumor cell line (Figure 5d).

We then investigated the effect of grafting PMSEA to IONPs on their cellular uptake. Both IONP@PMSEA and IONP@PEGA particles were incubated with Raw 264.7 and MDA‐MB‐468 for up to 24 h followed by Prussian Blue Staining experiments. As shown in Figure 6a, the PEGA‐grafted particles displayed a significantly increased cellular uptake compared with the PMSEA‐grafted counterparts for the macrophage cell line Raw 264.7 after 6 and 24 h of incubation. As expected, the tumor cell uptake of the IONPs was significantly lower than that by macrophage cells. However, a relatively lower uptake of PMSEA‐grafted nanoparticles than their PEGylated counterparts was also observed. This was further confirmed by a quantitative measurement of the iron content taken up by the cells using inductively coupled plasma optical emission spectroscopy (ICP‐OES) (Figure 6b,c). As shown in Figure 6b, for Raw 264.7 cells, the iron content per cell was measured to be 0.133 and 2.84 pg for IONP@PMSEA and IONP@PEGA particles, respectively, after 6 h incubation; and 0.73 and 12.04 pg, respectively, after 24 h incubation, indicating a remarkably reduced cellular uptake (16.5 folds lower) due to the PMSEA coating. Collectively, the remarkably decreased macrophage cellular uptake as well as the reduced interaction with protein molecules according to the proteomic analysis suggest a superior “stealth” behavior of the PMSEA surface coating.

### 2.7 Pharmacokinetic Study of PMSEA‐Grafted IONPs

**Figure 7:**
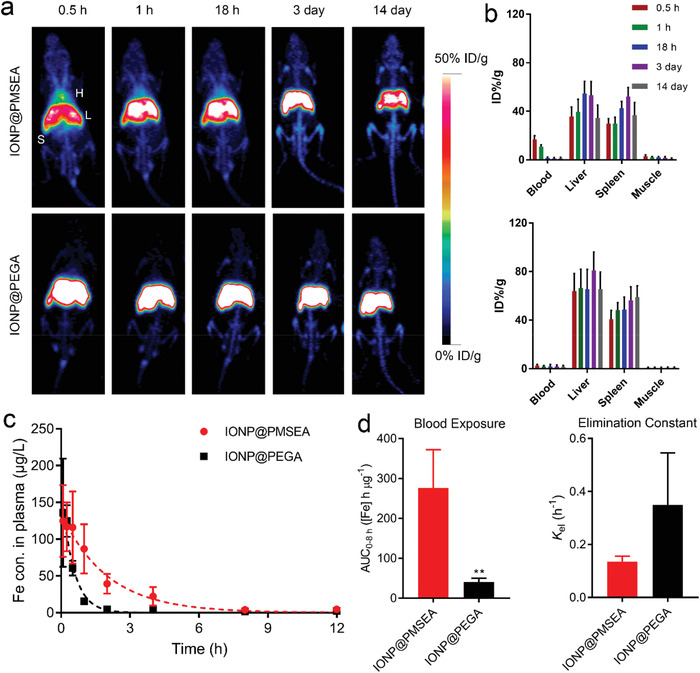
a) PET imaging of mice after administration of ^89^Zr‐IONP@PMSEA (upper) and ^89^Zr‐IONP@PEGA (lower) nanoparticles at different time points; H, heart; L, liver; S, spleen; b) Quantification of ^89^Zr‐IONP@PMSEA (upper) and ^89^Zr‐IONP@PEGA (lower) in the blood, liver, spleen, and muscle at various time points (*n* = 4, mean ± s.d.); c) Plasma concentrations of IONP@PMSEA and IONP@PEGA in SD rats; d) Pharmacokinetic analysis of blood exposure (AUC_0‐8 h_) and elimination constant (*K*_el_) of IONP@PMSEA and IONP@PEGA.

The in vivo behavior of the IONPs was also investigated by positron emission tomography (PET) for highly sensitive, quantitative, and noninvasive imaging. The IONPs were chelator‐free radiolabeled with a radionuclide ^89^Zr according to our previous work.^[^ ^23^ ^]^ Typically, the IONPs were incubated with ^89^Zr for 2 h at 75 °C and a 60% yield was achieved. To investigate the in vivo fate of the particles, ^89^Zr‐IONP@PMSEA and ^89^Zr‐IONP@PEGA particles with a normalized radiolabeling concentration were injected into healthy BALB/c mice, which were monitored for up to 14 days. As shown in **Figure** **7**a, PET signals due to both ^89^Zr‐IONP@PMSEA and ^89^Zr‐IONP@PEGA particles were primarily found in the liver of the mice.

In addition, the PET signals of ^89^Zr‐IONP@PMSEA particles were still present in the heart of the mice at 18 h post‐injection whereas the PET signals of ^89^Zr‐IONP@PEGA particles were not observed even at 0.5 h post‐injection. Moreover, the liver and spleen uptake of IONP@PMSEA was remarkably lower than for the IONP@PEGA particles (Figure 7b).

A pharmacokinetic (PK) study in Sprague Dawley (SD) rats after intravenous administration of IONPs was further conducted. The Fe concentration in plasma was determined by ICP‐OES. As shown in the PK profiles in Figure 7c,d, consistent with the results of the biodistribution study by PET imaging, the IONP@PMSEA particles showed a longer blood circulation compared with the IONP@PEGA particles evidenced by an almost sevenfold increase in the area under the plasma concentration versus time curve (AUC_0‐8 h_) (Figure 7d). The increase in blood exposure of IONP@PMSEA reflected the early confinement in the blood compartment. The apparent first‐order terminal elimination constants (*K*_el_) of particles were estimated by linear least‐squares regression on the semilog plot of the plasma concentration of particles versus time with the last three to four points of the curve. The result showed that the IONP@PEGA particles possessed a much larger *K*_el_ (0.35 ± 0.20) than the IONP@PMSEA particles (*K*_el_ ≈ 0.13 ± 0.02). The terminal elimination half‐lives (*t*_1/2_) of the IONP@PMSEA and IONP@PEGA particles were further calculated to be 5.15 and 1.98 h, respectively. These results collectively further demonstrated that the coating of PMSEA polymer largely improved the blood circulation time of nanoparticles compared with the PEGA coating by reducing interactions with the MPS.

## 3 Conclusion

In summary, we have demonstrated a novel surface modification of magnetic nanoparticles using a brush sulfoxide‐containing polymer prepared by RAFT polymerization. The resultant particles possessed excellent colloidal stability under physiological conditions. As a consequence of its remarkably hydrophilic nature, PMSEA provided a much enhanced “stealth” surface to the nanoparticles than the conventional brush PEGA polymers. Specifically, the coating of PMSEA reduced the interactions of IONPs with macrophages while significantly improved the toxicity profile of nanoparticles in comparison with the coating of PEGA. In addition, the coating of PMSEA greatly mitigate the interaction of IONPs with proteins. Importantly, the very few unique proteins associated with IONP@PMSEA participated no specific functional pathways related to immune response while the unique proteins associated with IONP@PEGA were largely associated with pathways altering cell immune responses, transport and localization. The PMSEA‐coated IONPs displayed a much‐extended blood circulation time and reduced accumulation in the MPS system than the PEG‐coated particles, as indicated by the biodistribution and pharmacokinetic characterizations. However, in this work, the grafting densities of polymers on nanoparticles are different, which may also have some effects on the interaction of the nanoparticles with biological system. More detailed research on investigating the effect of grafting density is in progress to further demonstrate the efficacy of PMSEA as an alternative low‐fouling polymer. Collectively, our results highlight the superior low‐fouling property of PMSEA polymer, which has demonstrated huge potential as a remarkable coating material for nanoparticles. It is anticipated that this innovative polymeric material will gain versatile biological applications, and will particularly contribute to developing long‐circulating nanoparticles with improved delivery efficiency for advanced therapeutics and diagnostics.

## 4 Experimental Section

##### Chemicals

The chemicals and solvents for the syntheses of brush PMSEA and PEGA polymers were purchased from Sigma‐Aldrich and used as received. MSEA monomer was synthesized according to our previous work.^[^ ^15a^ ^]^ Iron oxide nanocrystals with oleic acid coating were purchased from Ocean NanoTech. Maleimide‐Cy5 was purchased from Lumiprobe Corp. Dulbecco's Modified Eagle Medium (DMEM) culture medium and fetal bovine serum (FBS) were obtained from Gibco (Grand Island, NY, USA). Raw 264.7 and MDA‐MB‐468 cells from American Type Culture Collection (Manassas, VA) were used as received. Plasma was prepared by collecting the top layer following centrifugation of fresh blood at 900 *g*, for 15 min, without brake. (Collected from a healthy human volunteer into Greiner Bio‐One sodium heparin VACUETTE blood collection tubes, in accordance with the University of Melbourne Human ethics approval 1443420 and the Australian National Health and Medical Research Council Statement on Ethical Conduct in Human Research.)

##### Synthesis of Diphosphonate‐Terminated PEGA and PMSEA Polymers

Typically, 28.4 mg (0.06 mmol) diphosphonate‐CTA, 1 mg (0.006 mmol) AIBN and 0.5 g (3 mmol) MSEA monomer were mixed in 2 mL of DMF followed by degassing with nitrogen for 30 min. The polymerization was conducted at 70 °C for 16 h. The polymer was purified by precipitation in diethyl ether for 3 times and dried under vacuum.

The monomer conversion was calculated by comparing the integral of ester bond of the monomer before and after polymerization at 4.51 and 4.38 ppm for PMSEA, and 4.38 and 4.15 ppm for PEGA, respectively. The degree of polymerization was calculated by ^1^H NMR by comparing the integral of the peak at 0.89 ppm due to terminal methyl group of the RAFT agent and the peak due to methylene next to the monomer ester bond of the polymer at 4.38 ppm for PMSEA and 4.15 ppm for PEGA. The molecular weight given by SEC was apparent molecular weight.

##### Size Exclusion Chromatography

Size Exclusion Chromatography (SEC) analyses of polymer samples were performed in *N*,*N*‐dimethylacetamide (DMAc with 0.03% w/v LiBr and 0.05% 2,6‐dibutyl‐4‐methylphenol (BHT) using a Shimadzu modular system comprising a DGU‐12A degasser, an SIL‐10AD automatic injector, and a 5.0 µm bead‐size guard column (50 × 7.8 mm^2^) followed by four 300 × 7.8 mm^2^ linear Phenogel columns (bead size: a 5.0 µm; pore sizes: 105, 104, 103, and 500 Å) and an RID‐10A differential refractive‐index detector. The temperature of columns was maintained at 50 °C using a CTO‐10A oven, and the flow rate was kept at 1 mL min^−1^ using a LC‐10AT pump. A molecular weight calibration curve was produced using commercial narrow molecular weight distribution polystyrene standards with molecular weights ranging from 500 to 106 g mol^−1^. Polymer solutions at 2−3 mg mL^−1^ were prepared in the eluent and filtered through 0.45 µm filters prior to injection.

##### Quantitative Analysis of Polymer Uptake Using Operetta

Raw 264.7 (2 × 10^4^ cells per well) and MDA‐MB‐468 (1 × 10^4^ cells per well) were seeded on 96 well plates and plated at 37 °C in a humidified incubator with 5% CO_2_ for 24 h. The cell uptake of the Cy5 labeled PMSEA and PEGA polymers was detected using an Operetta High‐Content Imaging System (PerkinElmer) equipped with a 10 ×/Olympus U Plan FLN, 0.3 NA. Fluorescence was visualized with the excitation at 620–640, emission 650–760. Data were automatically analyzed by determining the mean Cy5 fluorescence per well using Harmony High Content Imaging and Analysis software (v3.5.2). Data were expressed as the mean ± SD from three independent experiments. Statistics were computed with GraphPad Prism 7.01 using standard unpaired *t*‐test. A value of *p* < 0.05 was considered significant.

##### Cytokine Response to Polymers in Peripheral Blood Mononuclear Cells

Peripheral venous bloods were taken from three volunteers and peripheral blood mononuclear cells (PBMCs) were isolated by Ficoll–Hypaque density gradient centrifugation at room temperature. The PBMCs were cultured in DMEM supplemented with 10% fetal bovine serum, 1% of penicillin‐streptomycin solution at 37 °C and 5% CO_2_. A total of 5 × 10^5^ PBMCs per well on 96 well plate was exposed to the polymers and stimulating reagent, a mixture of PMA (50 ng mL^−1^) and ionomycin (250 ng mL^−1^) for 20 hours. Cell culture medium was set up as a negative control. The supernatants were collected and analyzed simultaneously for 8 cytokines, including IL‐2, IL‐4, IL‐5, IL‐6, IL‐10, IL‐17A, TNF‐*α*, and IFN‐*γ* with a multi‐analyte flow assay kit (LEGENDplex Human Th1/Th2 Panel (eight‐plex) with Filter Plate) according to the manufacturer's instructions (Cat # No: 740729, Biolegend, USA).

##### General Procedure of Grafting of Polymer on IONPs

10 mg of the purified iron oxide particles capped with oleic acid and 100 mg of PMSEA or PEGA polymers were dissolved in 5 mL of THF. The ligand exchange reaction took place overnight at 40 °C. Then, the resulting PEGylated particles were precipitated by cyclohexane, washed with cyclohexane three times, and finally dried under vacuum at room temperature. The resultant particles were then dissolved in water and purified by ultrafiltration with an Amicon Ultra centrifugal filter (100 kD).

##### Nuclear Magnetic Resonance Spectra

^1^H and ^13^C Nuclear Magnetic Resonance (NMR) spectra were recorded on a Bruker AC400F (400 MHz) spectrometer. Chloroform‐d (CDCl_3_), and DMSO‐*d_6_* were used as the solvents, depending on the particular substance being analyzed.

##### Transmission Electron Microscope

Transmission Electron Microscope (TEM) images were obtained using a JEOL JEM‐2011 TEM. Energy‐dispersive X‐ray spectroscopy (EDS) maps were measured using JEOL JEM‐ARM200f scanning transmission electron microscope (STEM).

##### Dynamic Light Scattering

Hydrodynamic size of the particles was analyzed at 298.0 K using Nano ZS (Malvern) equipped with a solid state He−Ne laser (*λ* = 632.8 nm).

##### Relaxivity

Relaxivity of IONPs was performed on a Bruker BioSpec 94/30 USR 9.4 T small animal MRI scanner. The *T*_2_ relaxation times of the solution of IONPs were measured using the MSME sequence (VTE = 7.7–154.4 ms, TR = 3000 ms, FOV = 45 × 45 mm, FA = 90°, matrix = 256 × 256, measurement time = 12 min 48 s and 20 × 1 mm^2^ slices).

##### Tensiometer Measurement

The dynamic surface tension of the IONPs at the water–air and water−toluene interfaces was measured using the pendant drop method (OCA20, Dataphysics, Stuttgart). A syringe filled with a solution of IONP@PMSEA or IONP@PEGA and connected to a needle was fixed vertically with the needle immersed in the toluene phase. A small amount of the solution was injected from the syringe to form a drop. The variation of drop shape with time was captured by automated camera at particular time intervals, and the interfacial tension (*γ*) was estimated by data fitting using the Laplace−Young equation

(2) $$\;\gamma =\frac{\Delta \rho gd_{e}}{H}$$

where *Δρ* is the density difference between the liquid drop and its surrounding medium, *g* is the gravitational acceleration, *d_e_* is the largest horizontal diameter of the drop, and *H* is a function of *S_n_* (= *d_n_*/*d_e_*), in which *d_n_* is the horizontal diameter at a distance equal to *d_e_* (*n*/10) from the bottom of the drop. All experiments were performed at room temperature (23 ± 1 °C).

##### Hydrophobicity Study

Surface hydrophobicity was quantified by measuring the adsorption of the hydrophobic dye Rose Bengal on the IONPs at increasing surface area as described in a previous work.^[^ ^12b^ ^]^ The IONPs (0, 0.06, 0.12, and 0.18 mg mL^−1^) were incubated with constant Rose Bengal concentration (20 µg mL^−1^) for 3 h, followed by separation using an Amicon Ultra centrifugal filter (100 kD). The adsorption of Rose Bengal was acquired on a Shimadzu UV‐3600 UV−vis‐NIR spectrophotometer in quartz cuvettes of 10 mm path length.

##### Protein Corona Formation and Isolation

IONPs were mixed with human plasma at a mass ratio of 1:5 and incubated at 37 °C for 24 h at shaking conditions. The sample suspensions were centrifuged at 16 300 *g* for 15 min at room temperature (RT) to isolate the hard corona complexes and were washed thrice with 1 × PBS to remove unbound proteins. Isolated hard corona was resuspended in 2 × reducing loading dye, incubated at 95 °C for 5 min and spun down. The supernatant (20 µL) was resolved on an SDS‐PAGE gel (Mini‐ PROTEAN TGX, Biorad), stained using Instant Blue Stain (Expedion Ltd) and destained using MilliQ water.

##### In‐Gel Proteolytic Digestion, LC‐MS/MS Label‐Free Quantitation, Analysis, and Protein Informatics

The resolved region of the gel was cut and subjected to an in‐gel trypsin digestion procedure, as described previously.^[^ ^24^ ^]^ The extracted peptides were dried and resuspended in 20 µL of 2% acetonitrile (ACN) and 0.1% formic acid, and stored at −20 °C until analysis. LC‐MS/MS analysis was carried out as described previously,^[^ ^25^ ^]^ with minor modifications. Briefly, LC‐MS/MS was performed using Q Exactive HF Hybrid Quadrupole‐Orbitrap mass spectrometer. Samples were loaded at a flow rate of 15 µL min^−1^ onto a reversed‐phase trap column (75 µm × 2 cm) Acclaim PepMap media (Dionex) in 2% ACN, 0.1% tifluoroacetic acid (TFA). Peptides were then eluted from the trap column at a flow rate of 0.25 µL min^−1^ through a reversed‐phase capillary column (75 µm × 50 cm) (LC Packings, Dionex). The HPLC gradient was set to 128 min using a gradient that reached 30% of ACN after 93 min, then 34% of ACN after 96 min, 79.2% of ACN after 101 min and 2% after 108 min for a further 20 min. The mass spectrometer was operated in data‐dependent mode with 2 microscan fourier transform mass spectrometry scan events at 60 000 resolution (MS) over the *m*/*z* range of 375–1575 Da in positive‐ion mode, and up to 30 data‐dependent higher energy collision dissociated MS/MS scans.

Peptide sequences (and protein identity) were determined using MaxQuant software (version 1.6.0.1) by matching the human protein database (Homo sapiens, uniprot‐proteome_UP000005640.fasta) and label free quantification of identified proteins was then performed as previously described.^[^ ^24^ ^]^

Experiments were done in triplicate and proteins detected in at least two replicates (intensity > 0) were used for further analyses. Intensity was used to approximate the relative protein abundance between the different types of IONPs and protein abundance in each type individually. A student's *t*‐test was used to evaluate the significance of differences observed across the three independent replicates of IONP@PMSEA and IONP@PEGA and *p*‐values < 0.05 were considered.

The bioinformatics interaction network analysis tool STRINGdb^[^ ^26^ ^]^ was used to build a protein–protein interaction network using the significantly perturbed proteins. Connectivity was based on experimental, database and co‐expression evidence and a strict minimum interaction score (>0.7) was applied to limit false positive associations in the predicted network. The STRINGdb protein connectivity output was exported to Cytoscape 3.6^[^ ^27^ ^]^ and the ClusterONE algorithm was used to integrate and visualize relationships between proteins that were significantly perturbed in and IONP@PEGA versus IONP@PMSEA. Volcano plots and hierarchical clustering algorithms were run in Metaboanalyst.^[^ ^28^ ^]^ Hierarchical clustering analysis was developed using 101 differentially regulated proteins.

##### Cellular Uptake Study

IONPs were incubated with Raw 264.7 and MDA‐MB‐468 cell lines for the cellular uptake study. The IONPs stock solutions were diluted in 1 × PBS and added to the existing media. Doses of different samples were 0.1 mg mL^−1^ per well. A time‐course experiment for Prussian Blue Staining was carried out at incubation times of 6 and 24 h. After the incubation, the cells were washed with PBS for three times and stained for 20 min in a mixture solution composed of equal parts of 20% hydrochloric acid and 10% potassium ferrocyanide prepared immediately before use. A time‐course experiment for ICP‐OES analysis was carried out at incubation times of 6 and 24 h.

##### Cell Viability Assay

The Alarma Blue assay was used for the cell viability evaluation. Raw264.7 and MDA‐MB‐468 were grown in Dulbecco's Modified Eagle Media (DMEM) culture media with 10% Fetal Bovine Serum (FBS). For each cell line, 1.5 × 10^4^ cells per well were exposed to materials (25, 50, 100, 200, 400, and 800 µg mL^−1^) for 24 h in 96‐well plates, with the final volume of 100 µL. Cell culture medium was used as a control. After exposure, the suspensions were removed and the cells were incubated with 10% Alamar Blue (Invitrogen) for 4 h at 37 °C. A microplate reader (CLARIOstar, BMG LABTECH) was used to read the fluorescence at 560 nm excitation and 590 nm emission. Background values (10% Alamar Blue in cell culture medium) were subtracted from each well and the average fluorescent intensity of the triplicates was calculated to indicate cell viability.

##### Chelator‐Free Radiolabeling of IONPs

To evaluate in vivo circulation behavior and biodistribution of IONPs, ^89^Zr was used to radiolabel the IONPs for PET imaging by a chelator‐free method.^[^ ^29^ ^]^ Briefly, for ^89^Zr‐labeling, 100 µL of IONPs dispersed in HEPES buffer was directly mixed with 1 mCi (or 37 MBq) of ^89^Zr‐oxalate. The final pH value was adjusted to 7−8 with 1 m Na_2_CO_3_. After shaking for 2 h at 75 °C, ^89^Zr‐IONPs were collected by centrifugation and finally dispersed in PBS. ^89^Zr labeling yield was monitored and quantified by using thin layer chromatography (TLC) with subsequent autoradiography.

##### In Vivo PET Imaging of ^89^Zr‐IONPs

All animal studies were performed under a protocol approved by the University of Wisconsin Institutional Animal Care and Use Committee. The BALB/c mice were anesthetized and intravenously injected with 150 µL (≈200 µCi or 7.4 MBq) of ^89^Zr‐IONPs in PBS (*n* = 4). Serial PET scans were performed at various time points post‐injection (p.i.) from 0.5 h to 14 days. ROI analysis of each PET scan was conducted to calculate the percentage of injected dose per gram of tissue (%ID/g) in mouse organs, using vendor software (Inveon Research Workplace [IRW]) on decay‐corrected whole‐body images.

##### Pharmacokinetic Studies of IONPs in SD Rats

Male SD rats (200 ± 20 g) were supplied by the Qinglong Mountain Animal Center (Nanjing, China). All of the animal studies were conducted in accordance with the principles of Laboratory Animal Care and approved by the China Pharmaceutical University Animal Management and Ethics Committee. For the pharmacokinetic study, six healthy SD rats were randomly divided into two groups and treated with IONP@PMSEA and IONP@PEGA solution at a dose of 10 mg Fe kg^−1^ body weight. All formulations were administered intravenously through the tail vein and the blood samples were collected into heparinized tubes at 5, 15, and 30 min, 1, 2, 4, 8, 12, and 24 h. The blood samples (0.5 mL) were collected and centrifuged (Nr.12154 rotor, Sigma 3K30) at 2600 × *g* for 10 min at 4 °C to obtain the plasma. 200 µL of plasma was collected and stored (−30 °C) for further treatment and determination. In addition, blank plasma was also collected to deduct the effects of endogenous iron ions. The concentration of the Fe in plasma was determined by ICP‐OES.

##### Calculation of PK Parameters

PK data were treated by noncompartmental analysis of plasma Fe concentration versus time profiles. AUC_0‐8 h_ values were calculated by the trapezoidal method from 0 to 8 h. The apparent first‐order terminal elimination rate (*K*_el_) was estimated by linear least‐squares regression on the semilog plot of the plasma concentration versus time with the last 3 to 4 points of the curve; *t*_1/2_ was assessed as ln(2)/Kel.

## Conflict of Interest

The authors declare no conflict of interest.

## Supporting information

Supporting Information

Supporting Information
