# Supplementary material for: Sulfoxide‐Containing Polymer‐Coated Nanoparticles Demonstrate Minimal Protein Fouling and Improved Blood Circulation
Source: Adv Sci (Weinh). 2020 May 17;7(13):2000406. doi: 10.1002/advs.202000406 (PMC7341081; doi:10.1002/advs.202000406)
Supplement: Supplementary file 1 — Supporting Information [file ADVS-7-2000406-s001.pdf]

Supporting Information

Sulfoxide-Containing Polymer-Coated Nanoparticles Demonstrate Minimal Protein Fouling and Improved Blood Circulation

Ruirui Qiao, Changkui Fu, Yuhuan Li, Xiaole Qi, Dalong Ni, Aparna Nandakumar, Ghizal Siddiqui, Haiyan Wang, Zheng Zhang, Tingting Wu, Jian Zhong, Shi-Yang Tang, Shuaijun Pan, Cheng Zhang, Michael R. Whittaker, Jonathan W. Engle, Darren J. Creek, Frank Caruso, Pu Chun Ke, Weibo Cai, Andrew K. Whittaker, and Thomas P. Davis**

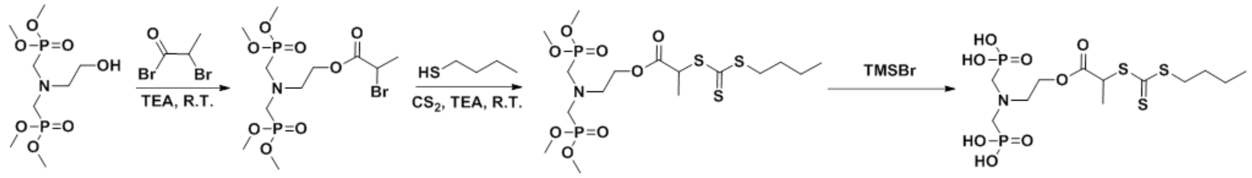

Figure S1. Synthesis of diphosphonate-terminated RAFT agent.

Synthesis of N,N-tetramethylbis(phosphonate)-2-hydroxylethyl-bis(methylene) amine. 60 g (0.982 mol) of 2-aminoethanol, 59 g (1.964 mol) of paraformaldehyde and 200 mL of THF were introduced in a two-necked flask. 213.2 g (1.964 mol) of dimethyl hydrogenophosphonate was then added in a dropwise manner and the reaction was conducted at 70 ^{o}C for 12 h. Afterwards, THF was removed by evaporation. The crude product was dissolved in chloroform and washed with a basic aqueous solution (0.1 N) of extract residual paraformaldehyde and dimethyl hydrogenophosphonate. After removal of chloroform, the product was obtained with 95% yield as a viscous orange liquid. ^{1}H NMR (400 MHz, Chloroform-*d*) δ 3.80 – 3.74 (m, 12H, $OC\underline{H}_3$), 3.64 – 3.59 (m, 2H, $CH_2C\underline{H}_2O$), 3.21 (d, *J* = 7.7 Hz, 4H, $NC\underline{H}_2P$), 2.93 (ddt, *J* = 6.3, 3.9, 1.3 Hz, 2H, $NC\underline{H}_2CH_2$).

2-(bis((dimethoxyphosphoryl)methyl)amino)ethyl 2-bromopropanoate. 4.9 g (11 mmol) of N,N-tetramethylbis(phosphonate)-2-hydroxylethyl-bis(methylene) amine and 2.4 mL (17 mmol) of TEA were mixed in 28 mL anhydrous DCM followed by dropwise addition of 1.8 mL (41 mmol) of 2-bromopropanoyl bromide under ice bath. The solution was left to react for 24 h. After removal of generated salts by filtration, the product was purified by passing

through a basic aluminum oxide column twice to remove excess 2-bromopropanoyl bromide, yielding a viscous liquid as the product. ^{1}H NMR (400 MHz, Chloroform-*d*) δ 4.37 (q, *J* = 6.9 Hz, 1H, $BrC\underline{H}CH_3$), 4.32 – 4.24 (m, 2H, $CH_2C\underline{H}_2O$), 3.82 – 3.74 (m, 12H, $OC\underline{H}_3$), 3.27 – 3.21 (m, 4H, $NC\underline{H}_2P$), 3.17 – 3.10 (m, 2H, $NC\underline{H}_2CH_2$), 1.82 (d, *J* = 7.0 Hz, 3H, $CHC\underline{H}_3$).

Synthesis of phosphonate-based RAFT agent. 4.0 g (44.4 mmol) butane-1-thiol was dissolved in 20 mL of water followed by addition of 4.5 g (44.4 mmol) of TEA under ice bath. After reacting for 30 min, 3.38 g (44.4 mmol) of carbon disulfide was added to the above solution in one portion. The solution turned yellow immediately and was left to react for another 4 h. Afterwards, 19.58 g (44.4 mmol) of 2-(bis((dimethoxyphosphoryl)methyl)amino)ethyl 2-bromopropanoate in 20 mL of acetone was added to the reaction, which was left at room temperature for 12 h while stirring. The product was purified by silica column (ethyl acetate/petroleum, v/v, 1:2), yielding a yellow liquid as the product. ^{1}H NMR (400 MHz, DMSO-d_6) δ 4.78 (q, *J* = 7.3 Hz, 1H, $SC\underline{H}CH_3$), 4.19 (dq, *J* = 7.9, 5.8 Hz, 2H, $CH_2C\underline{H}_2O$), 3.67 (dd, *J* = 10.5, 0.8 Hz, 12H, $OC\underline{H}_3$), 3.47 – 3.36 (m, 2H, $SC\underline{H}_2CH_2$), 3.25 – 3.17 (m, 4H, $NC\underline{H}_2P$), 3.01 (t, *J* = 5.7 Hz, 2H, $NC\underline{H}_2CH_2$), 1.64 (tt, *J* = 8.5, 6.8 Hz, 2H, $C\underline{H}_2CH_2CH_3$), 1.54 (d, *J* = 7.4 Hz, 3H, $C\underline{H}CH_3$), 1.45 – 1.31 (m, 2H, $CH_2C\underline{H}_2CH_3$), 0.90 (t, *J* = 7.4 Hz, 3H, $CH_2CH_2C\underline{H}_3$).

Deprotection of phosphonate-based RAFT agent. 200 mg of phosphonate-based RAFT agent was dissolved in 2 mL of DCM and degassed by nitrogen for 30 min. Then 582 μL of TMSBr in 1 mL of DCM was injected into the above solution. The reaction was kept at room temperature for 16 h. After that, the volatile components was removed by evaporation and the residues was dissolved in methanol and stirred at room temperature for another 2 h. After vacuum evaporation, the deprotected product was obtained as a yellow liquid. ^{1}H NMR (400 MHz, DMSO-d_6) δ 4.82 (q, *J* = 7.3 Hz, 1H, $SC\underline{H}CH_3$), 4.46 (q, *J* = 5.0 Hz, 2H, $CH_2C\underline{H}_2O$), 3.70 – 3.62 (m, 2H, $NC\underline{H}_2CH_2$), 3.61 – 3.52 (m, 4H, $NC\underline{H}_2P$), 3.39 (m, 2H, $SC\underline{H}_2CH_2$), 1.63

(m, 2H, C<u>H</u>$_2$CH$_2$CH$_3$), 1.56 (d, *J* = 7.3 Hz, 3H, C<u>H</u>CH$_3$), 1.42 – 1.31 (m, 2H, CH$_2$C<u>H</u>$_2$CH$_3$), 0.88 (t, *J* = 7.3, 3H, CH$_2$CH$_2$C<u>H</u>$_3$).

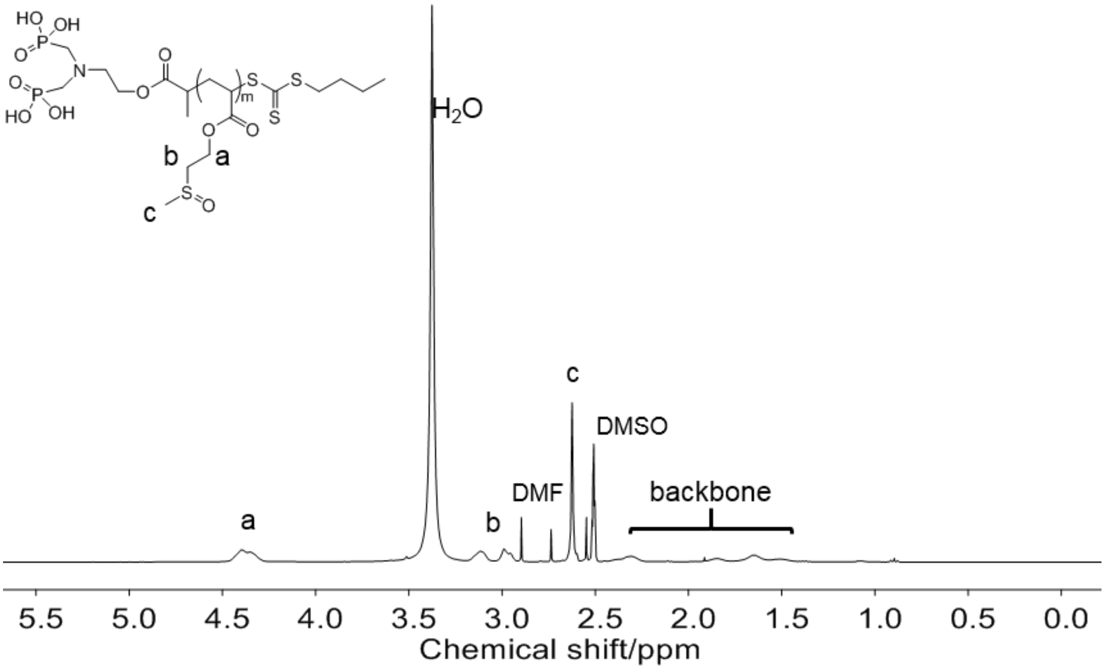

Figure S2. ^{1}H NMR (DMSO-d_6) of PMSEA polymer.

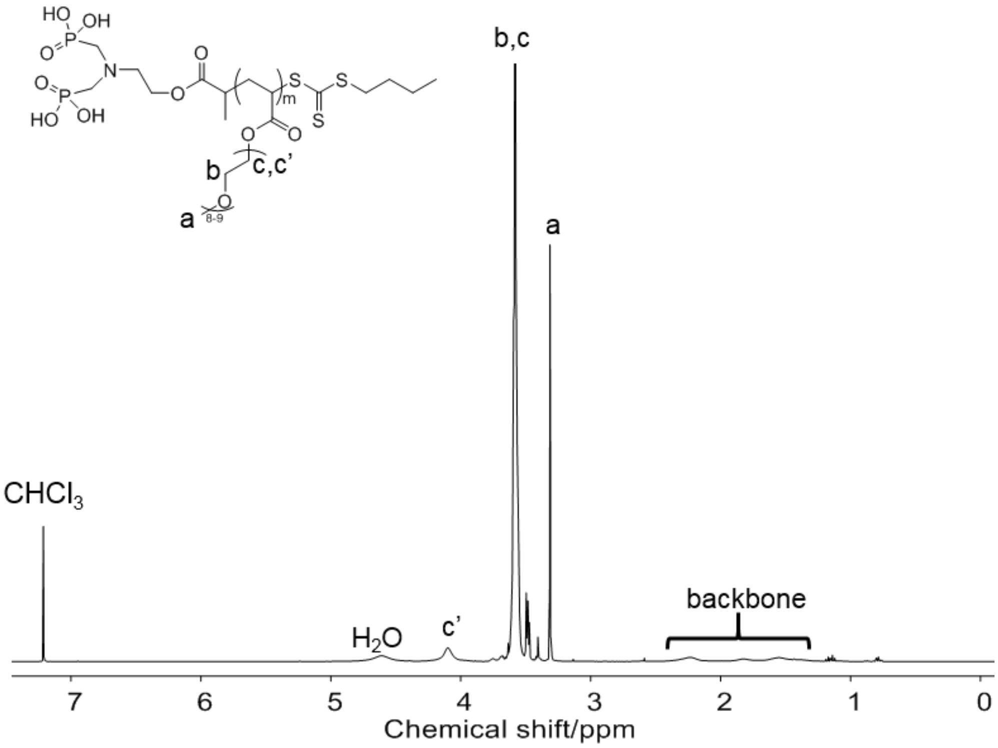

Figure S3. ^{1}H NMR ($CDCl_3$) of PEGA polymer.

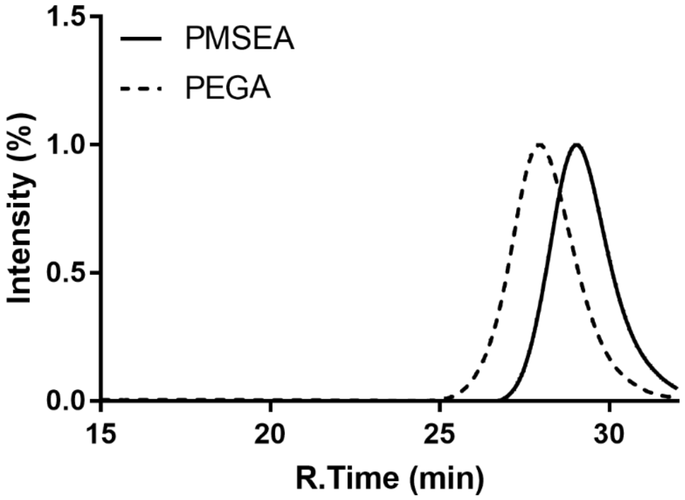

Figure S4. Retention time (as measured by SEC) of diphosphonate-terminated PMSEA and PEGA polymers.

Table S1. Details of PMSEA and PEGA polymers prepared by RAFT polymerization

Polymer	DP	Conv.	$M_{n,\ GPC}$ (g/mol)	$Đ_M$
PMSEA	50	88%	8762	1.29
PEGA	50	82%	16820	1.29

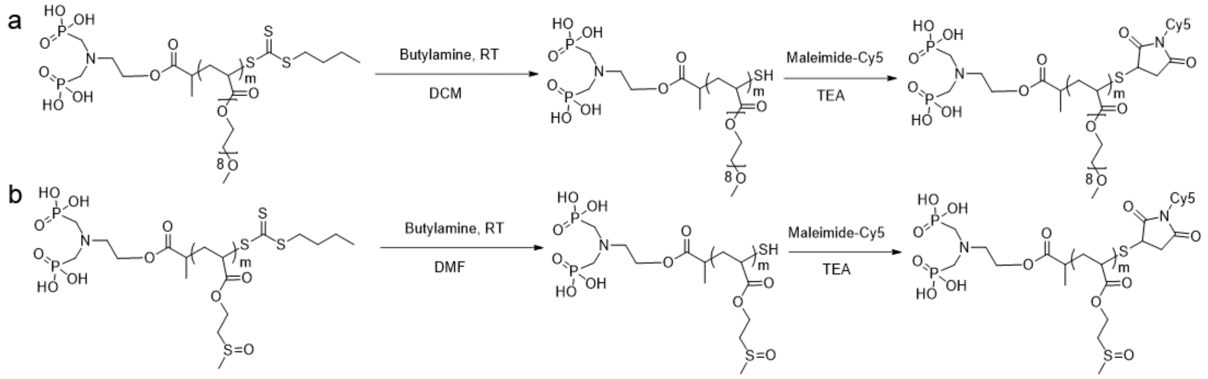

Figure S5. Cy5 labelling of diphosphonate-terminated a) PEGA and b) PMSEA polymers.

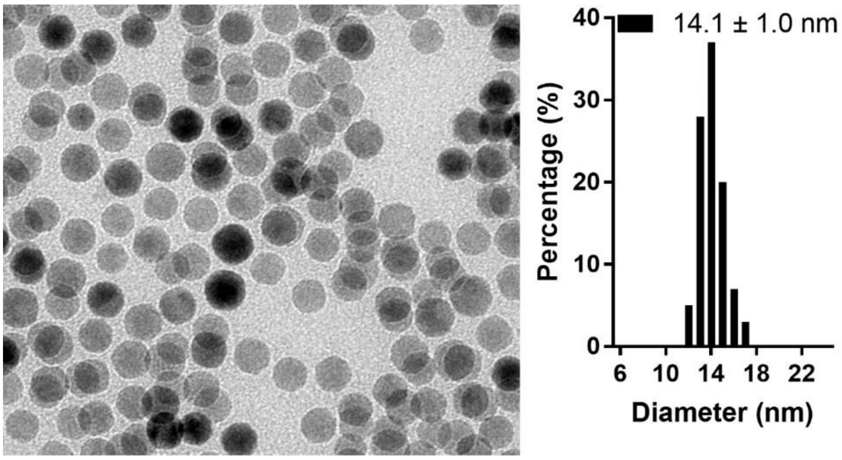

Figure S6. TEM and histography of IONP@OA particles.

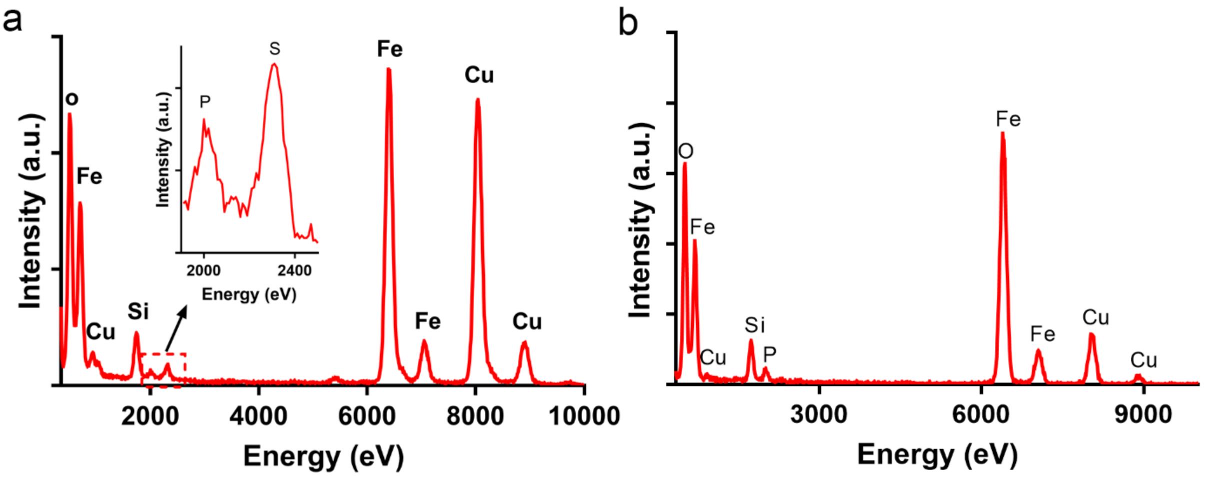

Figure S7. EDS spectrum of a) IONP@PMSEA and b) IONP@PEGA.

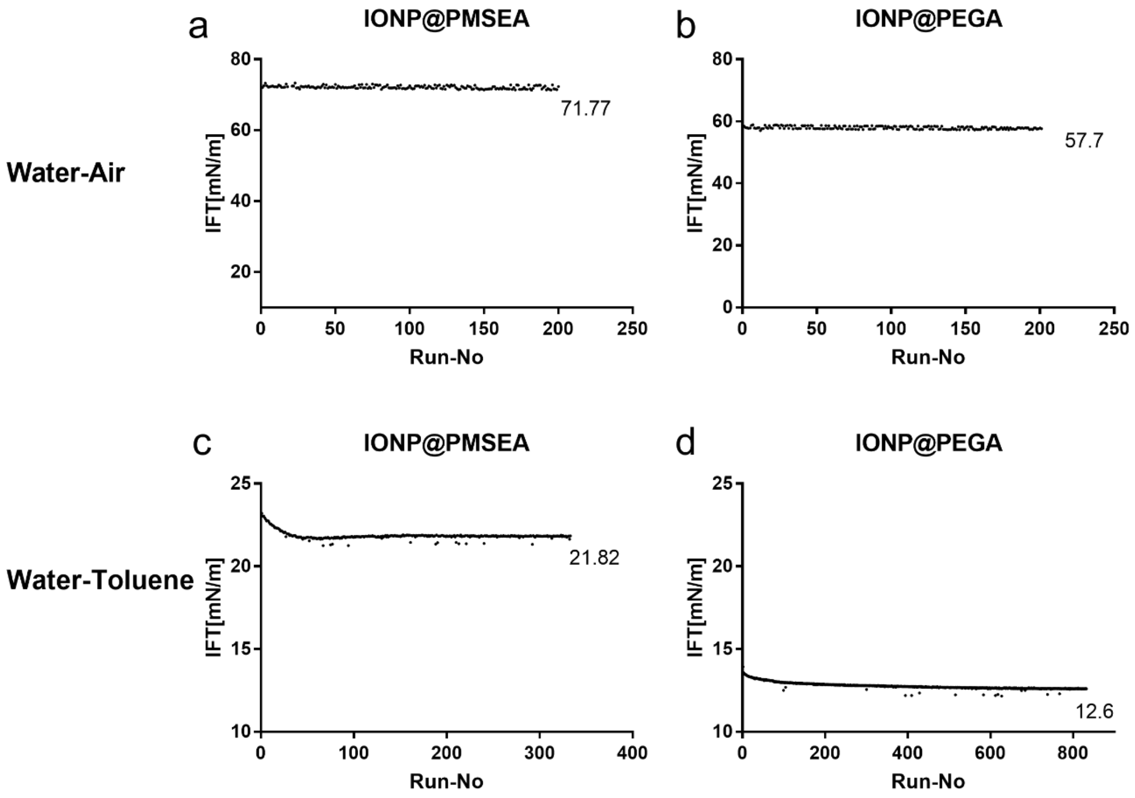

Figure S8. Time dependence of the interfacial tension of IONP@PMSEA and IONP@PEGA particles at the Water-Air (a and b) and Water–Toluene interface (c and d). The number near each plot indicates the average IFT value at the equilibrium run-No.

Table S2. Interfacial intension of IONP@PMSEA and IONP@PEGA

Nanoparticle	Interfacial Tension at Water-Air interface (mN/m)	Interfacial Tension at Water-Toluene interface (mN/m)
IONP@PMSEA	71.77±0.48	21.82±0.03
IONP@PEGA	57.7±0.2	12.6±0.01

Calculation of TSA. As shown in Table S3, the values of TSA defined here is referred to the surface area per particle ($4\pi r^2$) multiplied by the total number of NPs. The total number of NPs was derived from the total mass of NPs divided by the mass per particle, which corresponded to the product of the density of bulk gold (ρ_{core} = 5.17 g/cm^3) and the volume of a single particle ($4/3\pi r^3$). The radius of the NPs was measured from TEM and both the surface area and volume calculations require the assumption that the particle assumes a spherical shape.

Table S3. Total Surface Area (TSA) of nanoparticles (IONP@PEGA and IONP@PMSEA)

Volume of NPs solution (μL)	Mass of NPs (mg)	Total number of NPs	TSA (cm^2)
0			
30	0.18	1.05×10^{12}	52.5
60	0.36	2.09×10^{12}	105
120	0.72	4.18×10^{12}	210

Checklist

Minimum Information Reporting in Bio–Nano Experimental Literature

The MIRIBEL guidelines were introduced here: https://doi.org/10.1038/s41565-018-0246-4
The development of these guidelines was led by the ARC Centre of Excellence in Convergent Bio-Nano Science and Technology: https://www.cbns.org.au/. Any updates or revisions to this document will be made available here: http://doi.org/10.17605/OSF.IO/SMVTF. This document is made available under a CC-BY 4.0 license: https://creativecommons.org/licenses/by/4.0/.

The MIRIBEL guidelines were developed to facilitate reporting and dissemination of research in bio–nano science. Their development was inspired by various similar efforts:

- MIAME (microarray experiments): *Nat. Genet.* **29** (2001), 365; http://doi.org/10.1038/ng1201-365
- MIRIAM (biochemical models): *Nat. Biotechnol.* **23** (2005) 1509; http://doi.org/10.1038/nbt1156
- MIBBI (biology/biomedicine): *Nat. Biotechnol.* **26** (2008) 889; http://doi.org/10.1038/nbt.1411
- MIGS (genome sequencing): *Nat. Biotechnol.* **26** (2008) 541; http://doi.org/10.1038/nbt1360
- MIQE (quantitative PCR): *Clin. Chem.* **55** (2009) 611; http://doi.org/10.1373/clinchem.2008.112797
- ARRIVE (animal research): *PLOS Biol.* **8** (2010) e1000412; http://doi.org/10.1371/journal.pbio.1000412
- *Nature*'s reporting standards:
  - Life science: https://www.nature.com/authors/policies/reporting.pdf; e.g., *Nat. Nanotechnol.* **9** (2014) 949; http://doi.org/10.1038/nnano.2014.287
  - Solar cells: https://www.nature.com/authors/policies/solarchecklist.pdf; e.g., *Nat. Photonics* **9** (2015) 703; http://doi.org/10.1038/nphoton.2015.233
  - Lasers: https://www.nature.com/authors/policies/laserchecklist.pdf; e.g., *Nat. Photonics* **11** (2017) 139; http://doi.org/10.1038/nphoton.2017.28
- The "TOP guidelines": e.g., *Science* **352** (2016) 1147; http://doi.org/10.1126/science.aag2359

Similar to many of the efforts listed above, the parameters included in this checklist are **not** intended to be definitive requirements; instead they are intended as 'points to be considered', with authors themselves deciding which parameters are—and which are not—appropriate for their specific study.

This document is intended to be a living document, which we propose is revisited and amended annually by interested members of the community, who are encouraged to contact the authors of this document. Parts of this document were developed at the annual International Nanomedicine Conference in Sydney, Australia: http://www.oznanomed.org/, which will continue to act as a venue for their review and development, and interested members of the community are encouraged to attend.

After filling out the following pages, this checklist document can be attached as a "Supporting Information" document during submission of a manuscript to inform Editors and Reviewers (and eventually readers) that all points of MIRIBEL have been considered.

Supplementary Table 1. Material characterization*

Question	Yes	No
1.1 Are "**best reporting practices**" **available** for the nanomaterial used? For examples, see *Chem. Mater.* **28** (2016) 3535; http://doi.org/10.1021/acs.chemmater.6b01854 and *Chem. Mater.* **29** (2017) 1; http://doi.org/10.1021/acs.chemmater.6b05235		N/A
1.2 If they are available, **are they used**? If not available, ignore this question and proceed to the next one.		
1.3 Are extensive and clear instructions reported detailing all steps of **synthesis** and the resulting **composition** of the nanomaterial? For examples, see *Chem. Mater.* **26** (2014) 1765; http://doi.org/10.1021/cm500632c, and *Chem. Mater.* **26** (2014) 2211; http://doi.org/10.1021/cm5010449. Extensive use of photos, images, and videos are strongly encouraged. For example, see *Chem. Mater.* **28** (2016) 8441; http://doi.org/10.1021/acs.chemmater.6b04639	√	
1.4 Is the **size** (or **dimensions**, if non-spherical) and **shape of** the nanomaterial reported?	√	
1.5 Is the **size dispersity** or **aggregation** of the nanomaterial reported?	√	
1.6 Is the **zeta potential** of the nanomaterial reported?		√
1.7 Is the **density (mass/volume)** of the nanomaterial reported?	√	
1.8 Is the amount of any **drug loaded** reported? 'Drug' here broadly refers to functional cargos (e.g., proteins, small molecules, nucleic acids).		N/A
1.9 Is the **targeting performance** of the nanomaterial reported, including **amount** of ligand bound to the nanomaterial if the material has been functionalised through addition of targeting ligands?		N/A
1.10 Is the **label signal** per nanomaterial/particle reported? For example, fluorescence signal per particle for fluorescently labelled nanomaterials.		N/A
1.11 If a material property not listed here is varied, has it been **quantified**?	√	
1.12 Were characterizations performed in a **fluid mimicking biological conditions**?	√	
1.13 Are details of how these parameters were **measured/estimated** provided?	√	
Explanation for **No** (if needed):		

*****Ideally, material characterization should be performed in the same biological environment as that in which the study will be conducted. For example, for cell culture studies with nanoparticles, characterization steps would ideally be performed on nanoparticles dispersed in cell culture media. If this is not possible, then characteristics of the dispersant used (e.g.,

pH, ionic strength) should mimic as much as possible the biological environment being studied.

Supplementary Table 2. Material characterization*

Question	Yes	No
2.1 Are **cell seeding details**, including **number of cells plated, confluency at start of experiment,** and **time between seeding and experiment** reported?	√	
2.2 If a standardised cell line is used, are the **designation and source** provided?	√	
2.3 Is the **passage number** (total number of times a cell culture has been subcultured) known and reported?		√
2.4 Is the last instance of **verification of cell line** reported? If no verification has been performed, is the time passed and passage number since acquisition from trusted source (e.g., ATCC or ECACC) reported? For information, see *Science* **347** (2015) 938; http://doi.org/10.1126/science.347.6225.938		√
2.5 Are the results from **mycoplasma testing** of cell cultures reported?	√	
2.6 Is the **background signal of cells**/**tissue** reported? (E.g., the fluorescence signal of cells without particles in the case of a flow cytometry experiment.)	√	
2.7 Are **toxicity studies** provided to demonstrate that the material has the expected toxicity, and that the experimental protocol followed does not?		√
2.8 Are details of media preparation (**type of media, serum,** any **added antibiotics**) provided?	√	
2.9 Is a **justification of the biological model** used provided? For examples for cancer models, see *Cancer Res.* **75** (2015) 4016; http://doi.org/10.1158/0008-5472.CAN-15-1558, and *Mol. Ther.* **20** (2012) 882; http://doi.org/10.1038/mt.2012.73, and *ACS Nano* **11** (2017) 9594; http://doi.org/10.1021/acsnano.7b04855		N/A
2.10 Is characterization of the **biological fluid** (*ex vivo*/*in vitro*) reported? For example, when investigating protein adsorption onto nanoparticles dispersed in blood serum, pertinent aspects of the blood serum should be characterised (e.g., protein concentrations and differences between donors used in study).		N/A
2.11 For **animal experiments**, are the ARRIVE guidelines followed? For details, see *PLOS Biol.* **8** (2010) e1000412; http://doi.org/10.1371/journal.pbio.1000412	√	
Explanation for **No** (if needed):		

*For *in vitro* experiments (e.g., cell culture), *ex vivo* experiments (e.g., in blood samples), and *in vivo* experiments (e.g., animal models). The questions above that are appropriate depend on the type of experiment conducted.

Supplementary Table 3. Material characterization*

Question	Yes	No
3.1 For cell culture experiments: are **cell culture dimensions** including **type of well**, **volume of added media**, reported? Are cell types (i.e.; adherent vs suspension) and **orientation** (if non-standard) reported?	√	
3.2 Is the **dose of material administered** reported? This is typically provided in nanomaterial mass, volume, number, or surface area added. Is sufficient information reported so that regardless of which one is provided, the other dosage metrics can be calculated (i.e. using the dimensions and density of the nanomaterial)?	√	
3.3 For each type of imaging performed, are details of how **imaging** was performed provided, including details of **shielding, non-uniform image processing**, and any **contrast agents** added?	√	
3.4 Are details of how the dose was administered provided, including **method of administration, injection location, rate of administration,** and details of **multiple injections**?	√	
3.5 Is the methodology used to **equalise dosage** provided?	√	
3.6 Is the **delivered dose** to tissues and/or organs (in vivo) reported, as % injected dose per gram of tissue (%ID g^{-1})?	√	
3.7 Is **mass of each organ/tissue measured** and **mass of material** reported?		√
3.8 Are the **signals of cells/tissues with nanomaterials** reported? For instance, for fluorescently labelled nanoparticles, the total number of particles per cell or the fluorescence intensity of particles + cells, at each assessed timepoint.	√	
3.9 Are **data analysis details**, including **code used** for analysis provided?	√	
3.10 Is the **raw data** or **distribution of values** underlying the reported results provided? For examples, see *R. Soc. Open Sci.* **3** (2016) 150547; http://doi.org/10.1098/rsos.150547, https://opennessinitiative.org/making-your-data-public/, http://journals.plos.org/plosone/s/data-availability, and https://www.nature.com/sdata/policies/repositories	√	
Explanation for **No** (if needed):		

* The use of protocol repositories (e.g., *Protocol Exchange* http://www.nature.com/protocolexchange/) and published standard methods and protocols

(e.g., *Chem. Mater*. **29** (2017) 1; http://doi.org/10.1021/acs.chemmater.6b05235, and *Chem. Mater.* **29** (2017) 475; http://doi.org/10.1021/acs.chemmater.6b05481) are encouraged.
